# Controlled Release of Felodipine from 3D-Printed Tablets with Constant Surface Area: Influence of Surface Geometry

**DOI:** 10.3390/pharmaceutics15020467

**Published:** 2023-01-31

**Authors:** Kasitpong Thanawuth, Sontaya Limmatvapirat, Catleya Rojviriya, Pornsak Sriamornsak

**Affiliations:** 1 Pharmaceutical Biopolymer Group (PBiG), Faculty of Pharmacy, Silpakorn University, Nakhon Pathom 73000, Thailand; 2 Synchrotron Light Research Institute (Public Organization), Nakhon Ratchasima 30000, Thailand; 3 Academy of Science, The Royal Society of Thailand, Bangkok 10300, Thailand

**Keywords:** 3D-printed tablets, surface geometry, constant surface area, FDM 3D printing

## Abstract

In this study, 3D-printed tablets with a constant surface area were designed and fabricated using polylactic acid (PLA) in the outer compartment and polyvinyl alcohol and felodipine (FDP) in the inner compartment. The influences of different surface geometries of the inner compartment, namely, round, hexagon, square, and triangle, on drug release from 3D-printed tablets were also studied. The morphology and porosity of the inner compartment were determined using scanning electron microscopy and synchrotron radiation X-ray tomographic microscopy, respectively. Additionally, drug content and drug release were also evaluated. The results revealed that the round-shaped geometry seemed to have the greatest total surface area of the inner compartment, followed by square-shaped, hexagon-shaped, and triangle-shaped geometries. FDP-loaded 3D-printed tablets with triangle and hexagon surface geometries had the slowest drug release (about 80% within 24 h). In the round-shaped and square-shaped 3D-printed tablets, complete drug release was observed within 12 h. Furthermore, the drug release from triangle-shaped 3D-printed tablets with double the volume of the inner compartment was faster than that of a smaller volume. This was due to the fact that a larger tablet volume increased the surface area contacting the medium, resulting in a faster drug release. The findings indicated that the surface geometry of 3D-printed tablets with a constant surface area affected drug release. This study suggests that 3D printing technology may be used to develop oral solid dosage forms suitable for customized therapeutic treatments.

## 1. Introduction

Three-dimensional printing technology refers to the fabrication of three-dimensional objects or materials processed layer by layer from digitally created designs. This technology has received much attention in the various fields of architecture, automotive, engineering, biomedical, as well as pharmaceutical applications. Interestingly, the 3D-pharmaceutical dosage form, Spritam™ tablet, which treats seizures, was first approved by the FDA in August 2015 [1]. After the first approval, a large number of 3D-printed products containing drugs has been growing. There are numerous advantages of 3D printing innovation, including better drug-controlled release, less adverse effect risk, the ability to design high drug loading, and the capability of producing personalized dose products.

According to the American Society for Testing and Materials (ASTM), 3D printing innovation can be categorized into seven groups: binder jetting, direct energy deposition, material extrusion, material jetting, powder bed fusion, sheet lamination, and vat photopolymerization [2]. However, material extrusion has been widely used to create solid objects and selected to fabricate dosage forms in the pharmaceutical field because of its versatility. Fuse deposition modeling (FDM) is one example of material extrusion-based 3D printing. This technique is described as the process in which solid materials are melted by heating during the printing process. Finally, the desired objects are obtained. FDM creates any object layer by layer from the bottom to the top using heating and thermoplastic filaments [3]. In addition, the post-processing procedures after printing were not required. Therefore, the polymers used in the extrusion process require melting at a printing temperature. By utilizing FDM, thermoplastic polymers are commonly used for creating 3D objects or materials due to the use of high temperatures during the production process. For instance, acrylonitrile butadiene styrene (ABS) is used to produce matrix models, and polylactic acid (PLA) and polyvinyl alcohol (PVA) are used to produce tablets [4]. These polymers have been the most preferred substances for FDM printing. Thus, the physical properties of polymers play an important role in selecting materials for 3D printing.

Nowadays, several processes are required to produce the traditional pharmaceutical dosage forms, especially tablets, which need to be modified to achieve tablets with the desired properties. Recently, the processes involved in tablet fabrication have been adjusted by modifying several physical barrier patterns, mainly coatings of membranes and release matrices, either inner or outer of the tablets. However, there are some limitations in the tablets’ fabrication and development process, such as time-consuming and multiple modification steps. In some cases of changing the tablets (shape and size), new equipment or some modifications of the manufacturing facilities may be required. Three-dimensional printing technology has been developed to produce pharmaceutical products to address these problems.

As mentioned earlier, 3D printing technology is one of the promising choices for fabricating pharmaceutical dosage forms, especially tablets. The 3D-printed tablets fabricated from FDM have been introduced and developed into various types, including immediate, sustained, and time-released tablets. The 3D-printed tablets can be easily modified to achieve different drug release patterns by selecting the appropriate polymers, geometric design modification, and compartmentalizing the matrixes in the formulated products. Among these procedures to control drug release, a few research studies have been conducted regarding geometric design modifications [5]. Therefore, the objectives of this study were to produce an FDM filament comprising the model drug in a water-soluble polymer (i.e., PVA) and then to create the FDP-loaded 3D-printed tablets with different surface geometries (inner compartment) and constant surface area by printing PLA for the outer compartment. The relationship between surface geometric patterns of inner compartment and the dissolution behavior of the FDP-loaded 3D-printed tablets was also investigated.

## 2. Materials and Methods

### 2.1. Materials

Felodipine (FDP), an antihypertensive agent used for the model drug (BCS Class II, low solubility and high permeability, molecular weight (MW) of 384.26 g/mol [6]), was purchased from Xilin Pharmaceutical Raw Material Co., Ltd. (Jiangsu, China). PVA powder (Parteck^®^ MXP), specifically developed for application in hot-melted extrusion to increase solubility, was a gift from Merck (Darmstadt, Germany). Parteck^®^ MXP is a semi-crystalline polymer, MW 32,000 g/mol, 40–45 °C of glass transition temperature, 170 °C of melting temperature, decomposition temperature above 250 °C, and melt viscosity at D = 200 s^−1^: 345.3 ± 7.8 mPa.s [7,8]. PLA filament with a 1.75 mm diameter was purchased from Zhejian Flashforge 3D Technology Co., Ltd. (Zhejiang, China). Polysorbate 80 (Tween^®^ 80) was purchased from PanReac AppliChem (Barcelona, Spain).

### 2.2. Preparation of FDP-Loaded Filaments

The FDP-loaded PVA filament, comprising 5% *w/w* FDP and 95% *w/w* of PVA, was fabricated via a single-screw extruder (model WellzoomTM C desktop extruder, Shenzhen Mistar Technology Co., Ltd., Shenzhen, China) with a nozzle diameter of 1.75 mm at a temperature of 185 °C. The extruder’s screw speed was set to 12 rpm. The filament was stored in a desiccator, at room temperature, before printing.

### 2.3. Design and Fabrication of FDP-Loaded 3D-Printed Tablets

**Figure 1:**
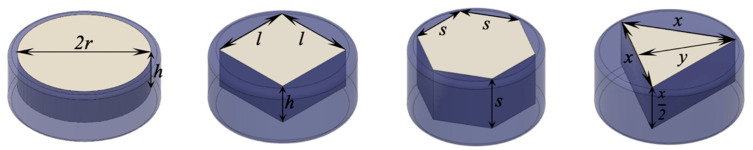
Design of FDP-loaded 3D-printed tablets with different internal surface geometries (left to right; round, square, hexagon, and triangle).

The controlled release of FDP-loaded 3D-printed tablets with two compartments was designed using Fusion 360 software (Autodesk Inc., San Rafael, CA, USA). The tablet models are illustrated in Figure 1. Various inner compartment geometries, including round, square, hexagon, and triangle, were designed. The inner tablet volume (mm^3^) was fixed at a constant value (Table 1). Based on our preliminary studies (data not shown), the volume of inner tablets that could adjust the FDP amount to the desired dose (5 mg/tablet) was 79.97 mm^3^. The outer compartment of all tablets was fabricated using PLA.

For the 3D-printing process, the model tablets were converted to gcode files using FlashPrint software version 3.28.0 (Zhejiang Flashforge 3D Technology Co., Ltd., Zhejiang, China). The 3D-printed tablets were produced on the Flashforge Creator Pro (Zhejiang Flashforge 3D Technology Co., Ltd., Zhejiang, China) with two printing nozzles. The outer compartment of the tablet, produced from commercial PLA filament, was printed using the right printing nozzle at 200 °C. During the inner compartment printing with the left printing nozzle, the FDP-loaded PVA filament was extruded into the nozzle at 195 °C. The other printing process settings were a bed temperature of 60 °C, a printing speed of 50 mm/s, a moving speed of 70 mm/s, a 100% infill density with a line pattern, and a layer thickness of 0.12 mm.

### 2.4. Characterization of FDP-Loaded 3D-Printed Tablets

#### 2.4.1. Morphological Characteristics of Tablets

The morphological characteristics of the FDP-loaded 3D-printed tablets were observed using the scanning electron microscopy (SEM) microscope (model Mira 3, Tescan, Brno, Czech Republic). All dried samples, consisting of whole tablets and cross-sectioned tablets using a cutter, were fixed on the SEM stub and then coated with gold layer. All samples were subjected to the SEM microscopy. The top and cross-section images of the tablets were photographed at the voltage of 5 kV with working distance of about 30 mm. The morphology of obtained tablets was analyzed.

#### 2.4.2. Differential Scanning Calorimetry (DSC)

The thermal properties of the materials, including FDP, PVA powder, physical mixtures of FDP and PVA, and FDP-loaded PVA filaments, were analyzed using the DSC 8000 (PerkinElmer, Waltham, MA, USA). Three to five mg of samples were placed and crimped in solid aluminum pans and heated from 20 to 250 °C with a heating rate of 10 °C/min. The nitrogen purge rate was set at 20 mL/min during the experiment. The data were analyzed with Pyris software.

#### 2.4.3. Thermogravimetric Analysis (TGA)

TGA was used to investigate the thermostability of the samples during filament extrusion and 3D-printing processes using a simultaneous thermal analyzer (model STA 6000, PerkinElmer, Waltham, MA, USA). For analysis, 3 to 5 mg of samples were weighted into open ceramic pans and heated from 35 to 600 °C at a rate of 10 °C/min under nitrogen purge (flow rate of 20 mL/min).

#### 2.4.4. Powder X-ray Diffractometry (PXRD)

The crystalline or amorphous behavior of all samples was investigated using the powder X-ray diffractometer (model MiniFlex II, Rigaku, Tokyo, Japan). The scanning conditions were a voltage of 30 kV, and a current of 15 mA with an angle in the range of 5 to 45°. The scanning speed was 4°/min using Cu-Kα radiation (0.154 nm).

#### 2.4.5. Synchrotron Radiation X-ray Tomographic Microscopy (SRXTM)

The porosity and pore surface area of the inner compartment of FDP-loaded 3D-printed tablets were analyzed using the Synchrotron XTM beamline (BL1.2 W: X-ray imaging and tomographic microscopy), Synchrotron Light Research Institute, Nakhon Ratchasima, Thailand. In this experiment, the inner compartment of tablets was unable to remain stable during direct exposure to synchrotron radiation. Therefore, the SRXTM imaging was performed using a polychromatic X-ray beam with a mean energy of 11.5 keV. The specimens were placed in the sample stage after being attached to the sample carrier. Then, X-ray tomography 3D imaging conditions were performed accordingly, where the 3D-printed tablet was rotated around an axis from 0 to 180° with an angular increment of 0.4°/s. For each sample measurement, a total of 500 projection images were gathered and then processed by means of correction, stitch, and reconstruction using the Octopus reconstruction software (Tescan, Gent, Belgium). The processed images were subjected to the Octopus analysis software (Tescan, Gent, Belgium) to examine the porosity. Afterward, Drishti software (National Computational Infrastructure, Canberra, Australia) was used to render the obtained 3D images.

### 2.5. Drug Content of FDP-Loaded Filaments and 3D-Printed Tablets

The FDP-loaded PVA filament and FDP-loaded 3D-printed tablets were evaluated for drug content using high-performance liquid chromatography (HPLC) (model Agilent 1100 series HPLC system, Agilent Technologies, CA, USA). Three different segments from the filament were collected to determine the drug content uniformity in the filament. In addition, 100 mg of each 3D-printed tablet was weighed and dissolved in 25 mL of 1% *w/v* polysorbate 80. The sample solutions were filtered (0.45-µm filter) and then injected (20 µL) into a 150 × 4.6 mm Luna 5u C18 column (Phenomenex, Cheshire, UK). The mobile phase composed of acetonitrile:methanol:phosphate buffer (40:20:40), was pumped at a flow rate of 1 mL/min with a control temperature of 35 °C. The phosphate-buffered solution was prepared by dissolving 6.9 g of monobasic sodium phosphate in water and adding 8 mL of 1 M phosphoric acid, then diluted with water until 100 mL. The absorbance was detected at a wavelength of 254 nm. All samples were analyzed in triplicate.

### 2.6. In Vitro Drug Release of 3D-Printed Tablets

The analysis method for the in vitro drug release of the FDP-loaded 3D-printed tablet was adapted from the protocol prescribed by the United States Pharmacopeia (USP 43-NF 38) for felodipine extended-release tablets [6]. The 750 mL of 1% *w/v* polysorbate 80 was added into the vessels in the USP dissolution apparatus II (model AT XtendTM, Sotax, Westborough, MA, USA). The FDP-loaded 3D-printed tablet was submerged by the medium on the vessel and the tests were run at 100 rpm paddle speed with a control temperature of 37 ± 0.5°C. The 3 mL of samples were withdrawn at various time intervals (45 min, 1, 2, 4, 6, 8, 10, 12, 14, 16, 18, 20, 22, and 24 h). Meanwhile, 3 mL of the fresh medium solution was added to maintain the sink condition. The drug concentrations were determined using HPLC (the method is described in Section 2.5.).

### 2.7. Mathematical Description of Drug Release

#### 2.7.1. Drug Release Kinetics Modeling

Six different kinetic models were considered to fit the observed data to determine the release behavior from the 3D-printed tablets. Model 1 is provided by the zero-order equation (Equation (1)).

(1) $$C_{t}=C_{0}-k_{0}t$$

where *C_t_* represents the drug amount released during the time *t*, *C_0_* is the initial concentration of drug release, and *k_0_* is the zero-order constant. For zero-order kinetics, the drug release is constant per unit of time. In other words, the drug release revealing zero-order kinetics depends on a time function, regardless of drug concentration.

Model 2 represents first-order kinetics. This kinetics type occurs when the drug is released proportionally per unit of time. First-order kinetics are concentration dependent. The model 2 equation is given by Equation (2) as follows.

(2) $$logC_{t}=logC_{0}-\left(\frac{k_{t}}{2.303}\right)$$

where *C_t_* represents the drug amount released during the time *t*, *C_0_* is the initial concentration of drug release, and *k_t_* is the first-order constant.

Model 3 is the Higuchi model. This model is often used to explain drug release from the inert matrix system. Equation (3) is illustrated the equation of the Higuchi model.

(3) $$C_{t}/C_{\infty }=k_{H}t^{1/2}$$

where *C_t_* and *C_∞_* are the drug release amounts at time *t* and infinite time, respectively, and *k_H_* is the release constant of Higuchi.

Model 4 is based on the Korsmeyer–Peppas model. The model is utilized to describe the drug release from the polymer matrix. Equation (4) is employed the Korsmeyer–Peppas kinetics equation.

(4) $$log\left(\frac{C_{t}}{C_{\infty }}\right)=log\;k+n\;log\;t$$

where *C_t_* and ***C****_∞_* are the cumulative drug release at time *t* and infinite time, respectively. *k* is a constant depending on the structure and geometrical characteristic of the system, and *n* is the exponent indicating the drug release mechanism. For designed FDP-loaded 3D-printed tablets, only one side of the tablet contacted the medium. Therefore, they might have a planar geometry system, and when *n* = 0.5, the drug release mechanism is the Fickian diffusion. An anomalous transport mechanism is observed when *n* is between 0.50 and 1.0. When *n* = 1.0, the drug release mechanism is Case II transport. Finally, if the value of *n* is more than 1, the drug release is the Super Case II model.

Model 5, the Hopfenberg model, Equation (5) creates a mathematical Hopfenberg model to predict drug release from surface-eroding polymers, providing a surface area that remains consistent during the degradation process [9].

(5) $$C_{t}/C_{\infty }=1-{\left[1-\left(k_{HB}t/C_{0}a_{0}\right)\right]}^{n}$$

where *C_t_* and *C_∞_* are the drug release amounts at time *t* and infinite time, respectively, *C_0_* is the initial drug release concentration, *k_HB_* is the erosion constant, *a_0_* is the half thickness of the film or the radius of a sphere or cylinder, and *n* is an exponent that changes with geometry, with *n* = 1, 2, and 3 for slab (flat), cylindrical, and spherical geometry, respectively [10].

Model 6 represents the Peppas–Sahlin model. This model is expressed to simultaneously describe two contribution mechanisms from the polymeric matrix (diffusional and relaxational). The Peppas–Sahlin kinetics equation is shown in Equation (6).

(6) $$C_{t}/C_{\infty }=k_{1}t^{m}+k_{2}t^{2m}$$

where *C_t_* and *C_∞_* are the drug release amounts at time *t* and infinite time, respectively, *k_1_* is the diffusion rate constant, *k_2_* is the relaxation rate constant, and *m* is the diffusion exponent.

All the above mathematical models are only valid in the 5 to 60% range of drug release from in vitro drug release. The experimental data for mathematical models of drug release were analyzed using the DDSolver program with Microsoft Excel software [11]. The correlation coefficient (R^2^) values and Akaike information criterion (AIC) were used to determine the best-fit model for drug release, with statistically higher R^2^ and lower AIC.

#### 2.7.2. Comparison of Drug Release Profiles

The two dissolution profiles were compared using similarity factors (*f_2_*). The similarity factor (*f_2_*) is described by an equation derived from the logarithmic reciprocal square root transformation of the sum of squared errors. This measurement was expressed as the percent (%) similarity of dissolution between the two curves [12], calculated by Equation (7):

(7) $$f_{2}=50\cdot log\left\{{\left[1+\left(\frac{1}{n}\right)\overset{n}{\underset{t=1}{\sum }}{\left|R_{t}-T_{t}\right|}^{2}\right]}^{-0.5}\times \;100\right\}$$

where *R_t_* and *T_t_* are drug release percentages at time points of reference and test sample, respectively, and *n* is the number of time points. The *f_2_* was calculated using the DDSolver software. The *f_2_* value is between 50 and 100, implying the similarity of the two release profiles. The similarity factors were computed in accordance with the best-fit model obtained from drug release kinetic analysis.

### 2.8. Stability Studies

FDP 3D-printed tablets with triangle surface geometry (TX, T2X (5% FDP), and T2X (10% FDP)) were placed in a desiccator chamber with 40% relative humidity control. The samples were stored for 12 months at ambient temperature (approximately 28 °C). Then, the drug content of FDP 3D-printed tablets was evaluated.

## 3. Results and Discussion

### 3.1. Preparation of FDP-Loaded Filaments

**Figure 2:**
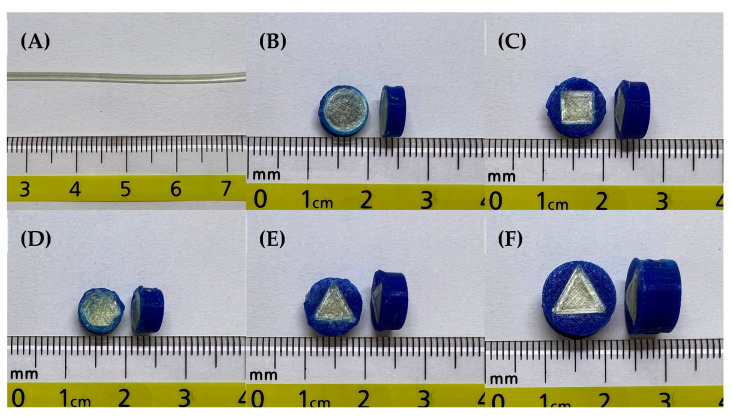
The photo images of (**A**) the FDP-loaded PVA filament, FDP-loaded 3D-printed tablets with different internal surface geometries, that is, (**B**) round, (**C**) square, (**D**) hexagon, and (**E**) triangle, and (**F**) FDP-loaded 3D-printed tablet with triangle surface geometry and double the volume of the inner compartment.

PVA, a water-soluble polymer, is often used for 3D printing because of its promising properties, including thermoplastic behavior and biodegradable properties. Therefore, it was selected to produce the filaments for 3D printing. The extrusion process was carried out at 185 °C with a 12 rpm extrusion speed. The result showed that the smooth surface with a uniform filament was successfully prepared by the abovementioned conditions. There were no defected along the fabricated filament (Figure 2A).

### 3.2. Design and Fabrication of FDP-Loaded 3D-Printed Tablets

As shown in Table 1, the tablet geometry variation revealed different surface areas despite having the same inner tablet volume. This was because the different patterns showed different formulas to achieve the required volume. For instance, at constant volume, the height values (h) of the tablet obtained from round and the other geometric designs were different, leading to the difference in calculated surface areas. It is clearly seen that the round-shaped tablet had the highest inner compartment surface area, followed by the square-shaped, hexagon-shaped, and triangle-shaped geometries, respectively. The results suggested that despite the same tablet volume, the total surface area of each tablet was different. The difference in total surface area would undoubtedly impact the release. The following section evaluated the drug release pattern to confirm the release pattern. The diameter and thickness of the obtained FDP-loaded 3D-printed tablets are also shown in Table 1.

### 3.3. Characterization of FDP-Loaded Filaments and 3D-Printed Tablets

#### 3.3.1. The Morphological Characteristics of Tablets

**Figure 3:**
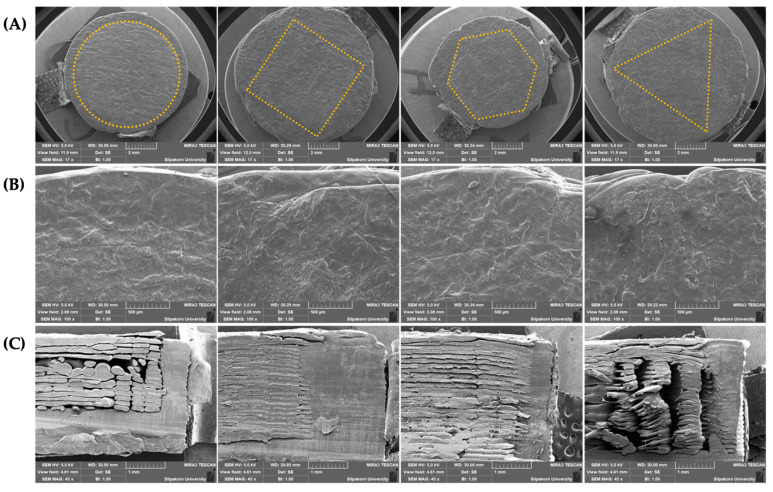
SEM images of FDP-loaded 3D-printed tablets with different internal surface geometries (from left to right: round, square, hexagon, and triangle), showing (**A**) a top view of the whole tablet, (**B**) a close-up view of the top surface, and (**C**) a cross-sectional view.

The photo images of FDP-loaded 3D-printed tablets are also displayed in Figure 2. All of the tablets were well-prepared without any defects. The SEM images of the FDP-loaded 3D-printed tablets with different internal surface geometries are shown in Figure 3. The tablets’ top surface was flat and relatively rough (Figure 3A,B). This is probably due to high printing temperature and high viscosity during both the extrusion and printing process [13]. Figure 3C depicts the inconsistent layer-by-layer printing pattern of the inner compartment of the 3D-printed tablets as a result of a printing process defect. Various infill shapes may affect the printing quality during the printing process because of the differences in the surface area to be printed.

Furthermore, the outer compartment in all of the tablets was densely compacted, making them extremely difficult to cut. As a result, this could explain why certain FDP-loaded 3D-printed tablets have a layering problem when viewed through an SEM.

#### 3.3.2. Differential Scanning Calorimetry

**Figure 4:**
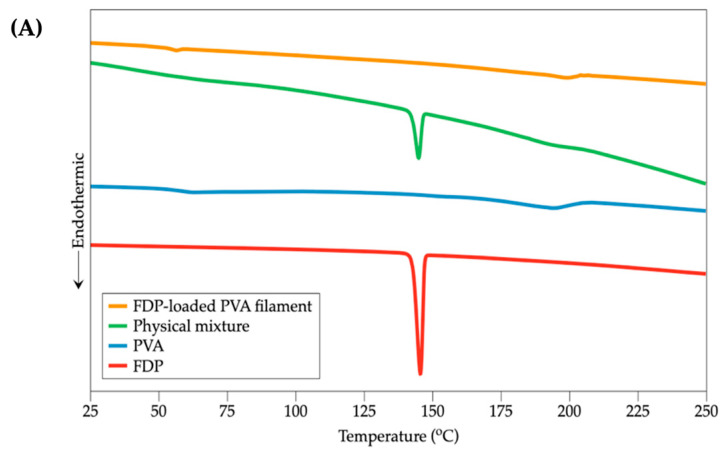

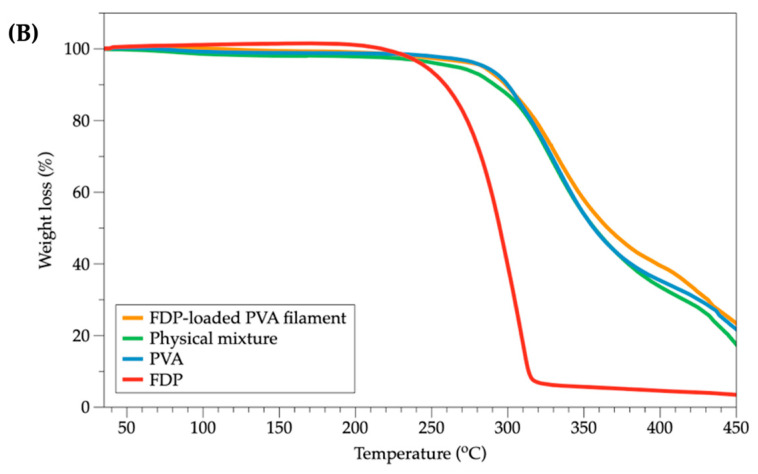
(**A**) DSC thermograms and (**B**) TGA thermograms of FDP, pure PVA, physical mixtures of FDP and PVA, and FDP-loaded PVA filaments.

The DSC thermograms of PVA, FDP, physical mixtures of FDP and PVA, and FDP-loaded PVA filaments are presented in Figure 4A. The PVA powder displayed glass transition endotherms at 57.3 °C and revealed a halo endothermic peak around 193.3 °C, suggesting the melting temperature. Furthermore, there was no sharp endothermic (melting) peak because of its semi-crystalline property. For FDP, the melting point was about 145.5 °C. In the physical mixture case, the melting point was similar to that of FDP. Nevertheless, the thermogram of the FDP-loaded PVA filament did not present the FDP melting peak, indicating the complete FDP incorporation into the PVA matrix [14].

#### 3.3.3. Thermogravimetric Analysis

TGA thermograms of PVA, FDP, physical mixtures of FDP and PVA, and FDP-loaded PVA filaments are depicted in Figure 4B. The TGA curve of PVA revealed a dramatic weight decrease with respect to its degradation temperature, at 293.6°C. FDP began to lose weight at approximately 261.4 °C. In addition, a slight weight loss change occurred during a process temperature increase. According to several reports, this may be due to water evaporation [15,16,17]. This result demonstrates that PVA and FDP were both stable at printing temperature (195 °C). The thermograms of PVA and FDP-loaded PVA filament almost overlapped, indicating that FDP was completely dispersed in the polymer matrix.

#### 3.3.4. Powder X-ray Diffractometry

**Figure 5:**
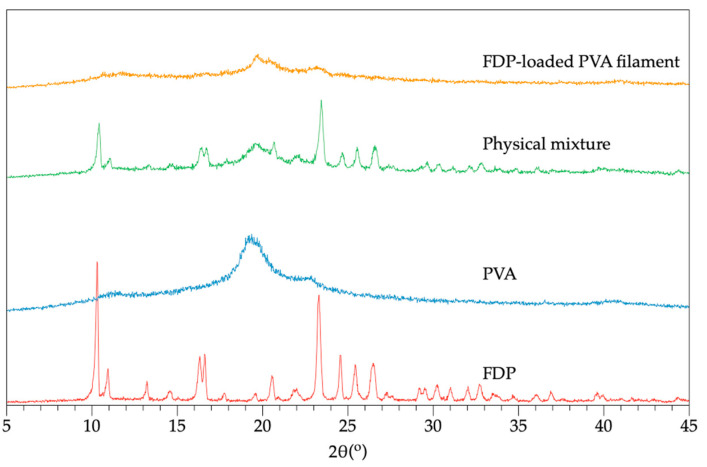
PXRD patterns of PVA, FDP, physical mixtures of FDP and PVA, and FDP-loaded PVA filaments.

PXRD patterns of PVA, FDP, physical mixtures of FDP and PVA, and FDP-loaded PVA filaments are displayed in Figure 5. The characteristic peak of PVA was observed at 19.41° [18]. Meanwhile, many distinctive peaks with high intensities appeared in the crystalline drug (FDP), including 10.31°, 10.93°, 16.31°, 16.60°, 21.90°, 24.56°, 25.43°, 26.46°, and 27.19° [19,20]. The combination of the characteristic peaks of PVA and FDP with a lower intensity signal was observed in the diffractogram of the physical mixture. Moreover, the corresponding crystalline peaks of the FDP completely disappeared in the FDP-loaded PVA filament, converting from a crystalline to an amorphous form. The result suggested that the FDP was entirely incorporated into the PVA matrix, enhancing the drug dissolution rate and solubility. The PXRD results were consonant with the DSC results, confirming the molecular dispersion of the FDP in the PVA matrix.

#### 3.3.5. Synchrotron Radiation X-ray Tomographic Microscopy

**Figure 6:**
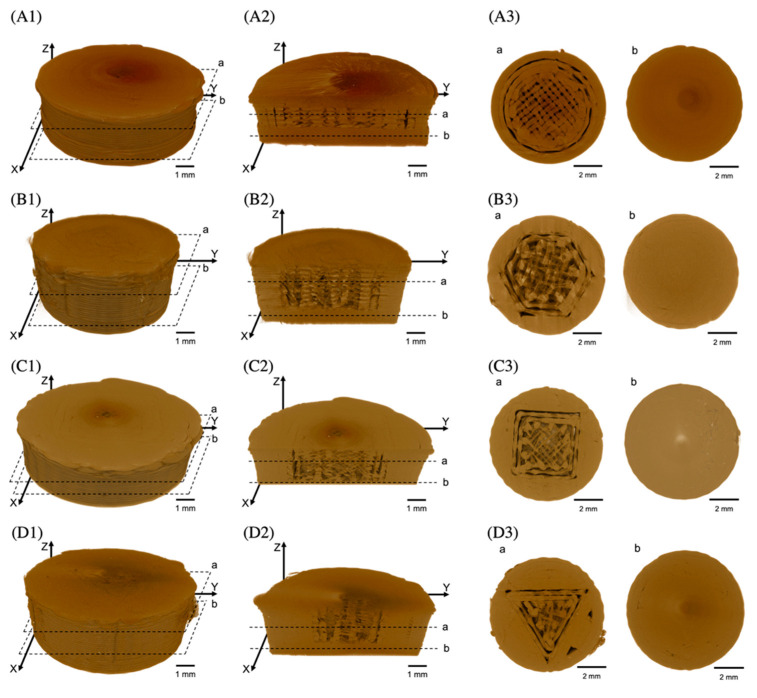
SRXTM images of FDP-loaded 3D-printed tablets with different inner surface geometries, showing (**A1**,**B1**,**C1**,**D1**) the entire tablet, (**A2**,**B2**,**C2**,**D2**) a vertical cross-section of the tablet, and (**A3**,**B3**,**C3**,**D3**) a horizontal cross-section of the tablet. The horizontal cross-section view images were created using the indicated positions (a and b).

**Figure 7:**
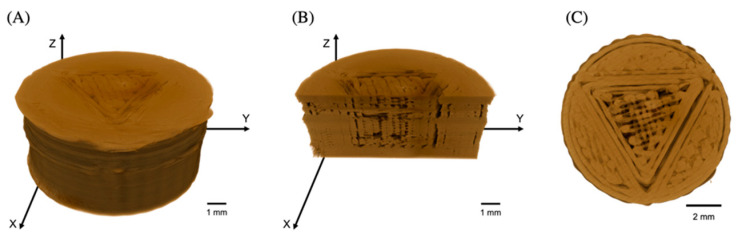
SRXTM images of FDP-loaded 3D-printed tablets with a triangle inner compartment (double the tablet volume, T2X), showing (**A**) the entire tablet, (**B**) a vertical cross-section, and (**C**) a horizontal cross-section of the tablet.

The X-ray micro-computed tomography (XµCT) technique has been an effective method to investigate the internal microstructure and the porosity of 3D objects, which was employed to perform more insightful studies on morphology and internal microstructure of the FDP-loaded 3D-printed tablets. The pore distribution in the inner compartment of FDP-loaded 3D-printed tablets was evaluated and then expressed as the porosity percentage. As shown in Table 2, the difference in geometric patterns exhibited different porosity values. The differences in porosity may be attributed to the fact that in the XµCT analyzing process, all detected pore types were calculated. Therefore, unexpected pores were also included in the porosity percentage measurements [21]. Additionally, for the FDM technique, pores can occur through several processes. In filament production, an uneven filament diameter provides several pores during the printing process [22,23]. When printing small objects, the FDM 3D printing tools may not function properly compared to larger objects [24]. According to SRXTM images, the % porosity of TX was higher than that of T2X because small tablets had a smaller surface area per layer, resulting in a shorter time for cooling before printing the next layer [24]. This problem might affect the material’s ability to partially solidify; moreover, some extruded material might be pulled during nozzle movement. For this reason, there were more pores between each layer on the smaller tablets. In other words, larger tablets had a larger surface area per layer as compared to TX tablets; therefore, they had more time to solidify before printing the next layer. Consequently, there were fewer noticeable gaps between the individual layers. According to Figure 6 and Figure 7, pore structures were observed in all tablets. However, the porosity detected in the outer compartment was also converted to a porosity percentage, yielding higher porosity values than expected.

#### 3.3.6. Drug Content of FDP Filament and 3D-Printed Tablets

The drug content is vital in defining the properties of the FDP-loaded 3D-printed tablets. The FDP contents in the filament and differences in the surface geometries of tablets were quantitatively determined. The drug loading percentage in the filament was 98.11 ± 1.22%, and in the tablets with different geometries, it was in the 94.68–98.36% range. This indicates that no drug degradation occurred due to the elevated temperature process during both the HME and printing processes [25]. TGA results, showing a negligible change in the FDP weight at printing temperature, also backed up this finding.

#### 3.3.7. In Vitro Drug Release of FDP-Loaded 3D-Printed Tablets

**Figure 8:**
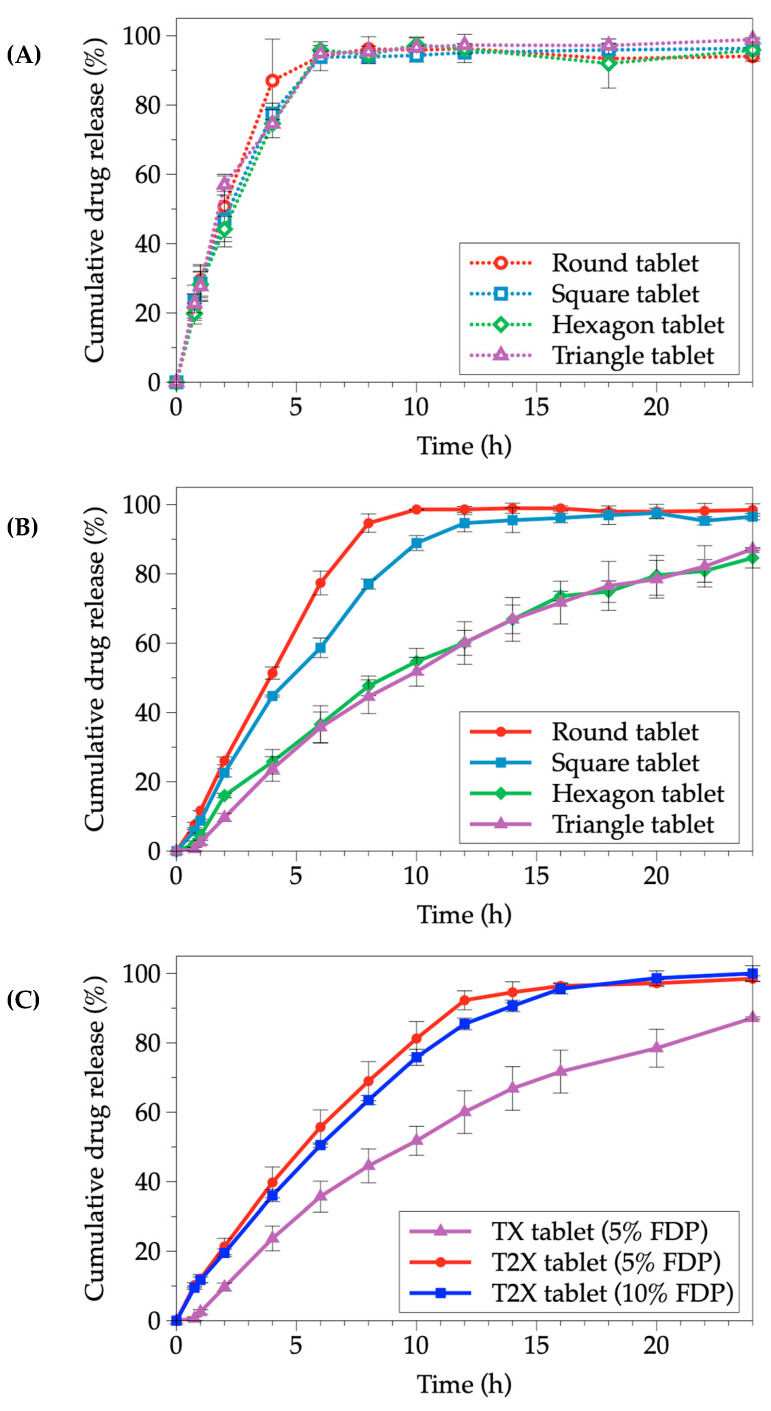
Cumulative drug release profiles of FDP-loaded 3D-printed tablets; (**A**) the tablets without an outer compartment, (**B**) the tablets with an outer compartment, (**C**) the tablets with triangle-shaped inner compartments having different inner compartment volumes or different drug concentrations.

Figure 8A depicts the drug release percentage from 3D-printed tablets without an outer compartment. As illustrated, the drug release percentage from the tablets without an outer compartment was not different in all geometric designs. After 6 h, all tablets had completely dissolved. The presence of the outer compartment caused a difference in drug release (Figure 8B); drug release from the round- and square-shaped inner compartments was completed within 12 h, indicating a slower drug release. The presence of caps, bases, or additional compartments is likely to affect drug release. However, after after 24 h of release testing, the drug release from the rest geometries, hexagonal- and triangle-shaped inner compartments, was approximately 80%. These findings were confirmed by Kardy and colleagues’ report [26]. The drug release from tablets with an outer compartment was release rate reduced. The reason could be that an outer structure played an important role in preventing dissolution medium penetration into an inner compartment, except from the top. As a result, water might diffuse into the tablets’ core through the cavities or pores generated from the printing process and existing in the core of tablet before the drug was dissolved and released from the tablets. The outer compartment presence would enable the development of controlled-release tablets. On the contrary, tablets without an outer compartment would be advantageous for manufacturing immediate-release tablets.

In general, increasing the drug concentration resulted in higher drug content and faster drug release [27]. As a result, we conducted a study with tablets of different volumes, TX and T2X (double the volume of TX), to examine the differences in drug release profiles. Figure 8C depicts the effect of tablet volume and the concentration of FDP loaded in the tablet on drug release profiles.

Thus, we performed a study using tablets containing different volumes, which were TX and T2X (double the volume, compared to TX), to examine the difference in drug release profiles. Figure 8C illustrates the tablet volume and concentration effects of the FDP loaded in the tablet on the drug release profiles. The results showed that increasing the tablet volume (from X to 2X) significantly increased cumulative drug release at the same amount of drug (5% FDP). Furthermore, 10% FDP (2X) was used to compare release profiles with 5% FDP in the same volume (2X). Therefore, the release profiles of both tablets were almost overlapped. As a result, the tablet volume or size revealed a significant factor in the drug release profile. Furthermore, the large surface area of the tablets in contact with the dissolution medium was reflected by the high volume of the tablets, resulting in a significant increase in the dissolution rate of the tablet with a large surface area.

Various mathematical models, including the zero-order, first-order, Higuchi, Korsmeyer–Peppas, Peppas–Shalin, and Hopfenberg models, were used to fit the experimental data with appropriate equations to predict the release patterns and behaviors of the FDP-loaded 3D-printed tablets. The Higuchi model was not appropriate for use in model fitting due to the inner compartment, composed of PVA polymer, not being an inert matrix, as evidenced by the swelling observed [28]. The R^2^ value obtained from each model was used to compare the fitted models’ quality. The release profiles of all inner-geometric designs followed the Peppas–Sahlin equation, as shown in Table 3 with R^2^ values ranging from 0.9977 to 0.9997. This model demonstrated the diffusion and relaxation of the drug release mechanism. The *k_2_* values were higher than the *k_1_*, indicating that the relaxation release mechanism was more prominent than the diffusion release mechanism for FDP-loaded 3D-printed tablets. Furthermore, as shown in Table 3, *k_1_* was extremely negative in all formulations, indicating that the Fickian diffusion mechanism less governed the drug release process. In other words, the diffusion processes of FDP-loaded 3D-printed tablets were virtually non-existent [29,30].

**Figure 9:**
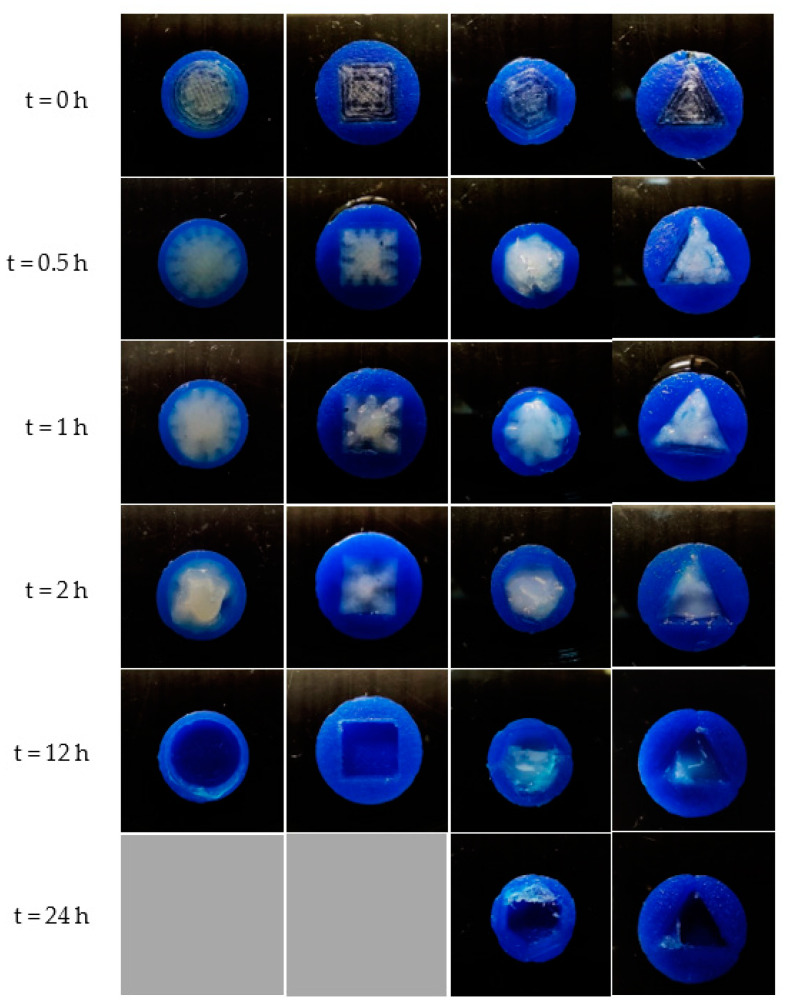
Top-view images of FDP-loaded 3D-printed tablets with round-, square-, hexagon-, and triangle-shaped inner compartments during the dissolution test.

Figure 9 displays the interior compartment with various surface geometries during the dissolution test. When exposed to the medium, the front surface of all 3D-printed tablets swelled, causing the polymer matrix of the inner compartment to dissolve. According to our previous reports [31], PVA, a water-soluble polymer obtained by the HME process, swelled when surrounded by the dissolution medium and then subjected to the erosion process [32].

#### 3.3.8. Comparison of Drug Release Profiles

The bootstrap *f_2_* tests of all FDP-loaded 3D-printed tablets were calculated to determine the similarity of the release patterns of the formulation with the different geometric designs. These results are displayed in Table 4. The findings indicated that *f_2_* obtained from the comparison between round- and square-shaped and between hexagonal- and triangle-shaped reached *f_2_* scores over 50, which were 51.82 and 76.51, respectively. It implied that a similar release profile pattern was observed in those geometric designs. In contrast, the *f_2_* values in other comparisons of other geometric designs were significantly lower than 50, indicating a lack of similarity. These findings indicated the insignificance of *f_2_* values due to the similarity of the surface area-to-volume ratio, as demonstrated by the *f_2_* values of round vs. square and hexagonal vs. triangular geometries. Therefore, geometric designs were crucial in determining drug release profiles [33,34].

#### 3.3.9. Stability Studies

The stability studies of FDP-loaded 3D-printed tablets with triangle surface geometry was evaluated under long-term conditions (28 °C/40%RH) for 12 months. The FDP content of TX, T2X (5% FDP), and T2X (10% FDP) tablets was found to be 4.01 ± 0.13 mg, 4.25 ± 0.16 mg, and 8.25 ± 0.05 mg, respectively. These results were then compared to the initial drug content immediately after the printing process (4.23 ± 0.03 mg of TX, 4.53 ± 0.36 mg of T2X (5% FDP), and 8.59 ± 0.17 mg of T2X (10% FDP)). As shown in Table 5, the drug content in the tablets before and after the stability studies was not significantly different (*p* > 0.05), as determined by a paired-samples t test. This finding indicated that the FDP-loaded 3D-printed tablet remained stable during storage.

## 4. Conclusions

The filaments fabricated by the hot-melt extrusion process were successfully prepared and used to fabricate FDP-loaded 3D-printed tablets. The FDP was completely incorporated into the PVA matrix, as confirmed by the measuring physico-chemical properties. The 3D printer could print the FDP-loaded 3D-printed tablets in various geometries, including round, triangle, square, and hexagon shapes. The drug release of FDP-loaded 3D-printed tablets was controlled by swelling rather than the Fickian diffusion process. Furthermore, drug release patterns from tablets primarily depended on the surface-to-volume ratio, with no dependence on the surface area. Dissolution patterns were similar in tablets with similar surface-to-volume ratios. Finally, fabricating 3D-printed tablets with different shapes and a specific surface-area-to-volume ratio may be able to regulate or control how the drugs are released. This could also be used to aid in the development of new dosage forms with specific applications.

## Figures and Tables

**Table 1:** Physical parameters for FDP-loaded 3D-printed tablets with an inner compartment volume constant.

Surface Geometry of Inner Tablet	Volume Equation	Inner Tablet Dimensions (mm)	Inner Tablet Volume (mm^3^)	Surface Area (mm^2^)	FDP-Loaded 3D-Printed Tablets	
					Diameter (mm)	Thickness (mm)
Round	*v* = ${\;\pi r}^{2}h$	*r* = 3.50, *h* = 2.00	79.97	38.48	8.23 ± 0.12	2.92 ± 0.01
Square	*v* = $\;l^{2}h$, *h* = $\frac{l}{2}$	*l* = 5.36, *h* = 2.38	79.97	28.72	9.26 ± 0.15	3.92 ± 0.07
Hexagon	*v* = $\frac{3\sqrt{3}}{2}s^{3}$	*s* = 3.09	79.97	24.88	7.53 ± 0.16	3.81 ± 0.01
Triangle (TX)	*v* = $\;\frac{x^{2}y}{4},$ *y* = $\frac{x\sqrt{3}}{2}$	*x* = 7.08, *y* = 6.13	79.97	21.73	9.12 ± 0.03	3.84 ± 0.05
Triangle (T2X)	*v =*$\;\frac{x^{2}y}{4},$*y* = $\frac{x\sqrt{3}}{2}$	*x* = 8.93, *y* = 7.73	159.94	34.49	11.53 ± 0.14	6.05 ± 0.10

Note: *v* represents the volume of inner compartment tablet, *r, h, l, s, x,* and *y* represent the dimensions of inner compartment tablet as shown in Figure 1.

**Table 2:** Porosity and pore surface area of inner compartments of FDP-loaded 3D-printed tablets using SRXTM.

Surface Geometry of Inner Tablet	Top Surface Area (mm^2^) = TSA	Pore Surface Area (mm^2^) = PSA	Total Surface Area (mm^2^) [TSA + PSA] = TA	Inner Tablet Volume = V	TA/V Ratio	Porosity (%)
Round	38.48	5.31 ± 1.27	43.79 ± 1.27	79.97	0.55 ± 0.02	5.37 ± 2.89
Square	28.72	12.29 ± 1.03	41.01 ± 1.03	79.97	0.51 ± 0.01	30.78 ± 4.30
Hexagon	24.88	9.52 ± 0.16	34.40 ± 0.16	79.97	0.43 ± 0.01	22.12 ± 1.00
Triangle (TX)	21.73	11.01 ± 0.71	32.74 ± 0.71	79.97	0.41 ± 0.01	14.68 ± 2.89
Triangle (T2X)	34.49	7.68 ± 1.96	42.17 ± 1.96	159.94	0.26 ± 0.01	5.83 ± 1.48

**Table 3:** Release kinetics of FDP-loaded 3D-printed tablets with different surface areas.

Model	Surface Geometry of Inner Tablet	R^2^	AIC	Parameters
Zero-order	Round	0.9982	11.0060	*k_0_* = 12.842
	Square	0.9894	24.6174	*k_0_* = 9.921
	Hexagon	0.9631	48.3447	*k_0_* = 5.247
	Triangle (TX)	0.9818	42.5807	*k_0_* = 5.097
	Triangle (T2X)	0.9800	26.4023	*k_0_* = 9.112
First-order	Round	0.9457	28.1522	*k_1_* = 0.189
	Square	0.9725	30.3707	*k_1_* = 0.151
	Hexagon	0.9937	32.4024	*k_1_* = 0.078
	Triangle (TX)	0.9864	39.3414	*k_1_* = 0.015
	Triangle (T2X)	0.9938	19.4127	*k_1_* = 0.135
Korsmeyer–Peppas	Round	0.9988	10.8932	*K* = 12.140, *n* = 1.036
	Square	0.9922	24.7875	*K* = 11.356, *n* = 0.926
	Hexagon	0.9866	41.2467	*K* = 8.475, *n* = 0.796
	Triangle (TX)	0.9953	31.8733	*K* = 1.894, *n* = 0.846
	Triangle (T2X)	0.9992	8.8577	*K* = 12.521, *n* = 0.825
Hopfenberg	Round	0.9985	12.2633	*k_HB_* = 0.132
	Square	0.9943	22.8770	*k_HB_* = 0.081
	Hexagon	0.9941	33.8798	*k_HB_* = 0.008
	Triangle (TX)	0.9918	37.3964	*k_HB_* = 0.030
	Triangle (T2X)	0.9986	12.5552	*k_HB_* = 0.044
Peppas–Sahlin	Round	0.9997	5.8503	*k_1_* = −11.427, *k_2_* = 22.922, *m* = 0.414
	Square	0.9981	18.2374	*k_1_* = −35.371, *k_2_* = 44.718, *m* = 0.272
	Hexagon	0.9977	27.2436	*k_1_* = −42.867, *k_2_* = 47.858, *m* = 0.204
	Triangle (TX)	0.9993	17.7354	*k_1_* = −34.537, *k_2_* = 37.006, *m* = 0.242
	Triangle (T2X)	0.9993	10.1858	*k_1_* = −3.153, *k_2_* = 15.494, *m* = 0.384

Note: R^2^ = correlation coefficient values, AIC = Akaike information criterion.

**Table 4:** Similarity factor (*f_2_*) results for dissolution profile comparison of FDP-loaded 3D-printed tablets.

Comparison	Similarity Factors (f2)	Interpretation
Round vs. square	51.82	Accept
Round vs. hexagon	32.48	Not accept
Round vs. triangle	30.89	Not accept
Square vs. hexagon	38.09	Not accept
Square vs. triangle	35.56	Not accept
Hexagon vs. triangle	76.51	Accept
Triangle (TX) vs. triangle (T2X)	39.47	Not accept

**Table 5:** Stability studies of 3D-printed tablets with triangle surface geometry.

Sample	Drug Content (mg)		Significance between before and after Stability Tests (Paired-Samples *t* Test)
	0 Month	12 Months	
TX	4.23 ± 0.03	4.01 ± 0.13	No
T2X (5% FDP)	4.53 ± 0.36	4.25 ± 0.16	No
T2X (10% FDP)	8.59 ± 0.17	8.25 ± 0.05	No

## Data Availability

Data are contained within the article.
